# The relationship between serum fibrosis markers and restrictive ventricular filling in patients with heart failure with reduced ejection fraction: A technetium-99m radionuclide ventriculography study

**DOI:** 10.18632/oncotarget.13795

**Published:** 2016-12-04

**Authors:** Yen-Tin Lin, Yen-Hung Lin, Xue-Ming Wu, Chi-Lun Ko, Ruoh-Fang Yen, Ying-Hsein Chen, Ron-Bin Hsu, Chi-Ming Lee, Shoei-Shen Wang, Ming-Fong Chen, Yen-Wen Wu

**Affiliations:** ^1^ Department of Internal Medicine, Taoyuan General Hospital, Ministry of Health and Welfare, Taoyuan, Taiwan; ^2^ Department of Internal Medicine, National Taiwan University Hospital and National Taiwan University College of Medicine, Taipei, Taiwan; ^3^ Department of Nuclear Medicine, National Taiwan University Hospital and National Taiwan University College of Medicine, Taipei, Taiwan; ^4^ Department of Surgery, National Taiwan University Hospital and National Taiwan University College of Medicine, Taipei, Taiwan; ^5^ Department of Medicine, Beth Israel Deaconess Medical Center/Harvard Medical School, Boston, Massachusetts, USA; ^6^ Cardiovascular Research Laboratory, Cardiovascular Center, Clinical Outcome Research and Training Center, Big Data Center, China Medical University Hospital, China Medical University, Taichung, Taiwan; ^7^ Cardiology Division of Cardiovascular Medical Center, Department of Nuclear Medicine, Far Eastern Memorial Hospital, New Taipei City, Taiwan; ^8^ National Yang-Ming University School of Medicine, Taipei, Taiwan

**Keywords:** fibrosis, restrictive filling, ventriculography

## Abstract

Myocardial fibrosis leads to a restrictive diastolic filling pattern of the left ventricle which is associated with a poor prognosis in patients with heart failure. We investigated the relationship between cardiac fibrosis and restrictive filling pattern of the left ventricle measured by Tc99m left ventriculography in patients with chronic symptomatic heart failure. Serum cardiac extracellular matrix markers including type I and III aminoterminal propeptide of procollagen (PINP and PIIINP), matrix metalloproteinase-2,9 (MMP-2,9), and tissue inhibitor of MMP-1 (TIMP-1) were analyzed. Fifty-one (39 males) patients were enrolled. Their median age was 51.8 years, and median left ventricular ejection fraction was 31.9%. Time to peak filling rate of the left ventricle was significantly correlated with serum levels of the three cardiac extracellular matrix markers (TIMP-1, PIIINP, and MMP-2). The patients with a restrictive diastolic filling pattern of the left ventricle (time to peak filling rate = 154 ms) had significantly higher levels of these extracellular matrix markers. In receiver operating characteristic curve analysis, areas under the curve of PIIINP, TIMP-1, and MMP-2 were 0.758, 0.695, and 0.751 to predict the presence of a restrictive pattern. In C-statistics, all three cardiac extracellular matrix markers significantly increased the area under the curve after adding creatinine. In net reclassification improvement and integrated discrimination improvement models, PIIINP and MMP-2 significantly improved the predictive power of age, creatinine and brain natriuretic peptide. In conclusion, serum extracellular matrix markers are significantly correlated with restrictive diastolic filling pattern of the left ventricle in patients with heart failure.

## INTRODUCTION

Heart failure (HF) is a major health problem affecting more than 23 million patients worldwide [1]. Despite remarkable improvements in medical treatment in recent decades, the prognosis of patients with HF remains poor [2].

Systolic function has been reported to be a prognostic predictor in HF with reduced ejection fraction (HFrEF) [3]. However, compared to the New York Heart Association functional classification, left ventricular ejection fraction (LVEF) has been reported to be a less reliable marker, especially in patients with atrial fibrillation [4, 5]. Beyond systolic function, abnormal diastolic function is also an important factor. Patients with HFrEF and concomitant diastolic dysfunction have been reported to have worse clinical outcomes, and especially those with severe impairment of diastolic function such as a restrictive filling pattern in mitral flow [6]. Many tools can estimate diastolic function, including echocardiography, cardiac magnetic resonance imaging (MRI), electrocardiography-gated myocardial perfusion single photon emission computed tomography (SPECT), and technetium 99m (Tc99m) left ventriculography. Echocardiography is simple, non-invasive and cost-effective, however it is limited by alterations in loading conditions, operator dependency, and echo window. Tc99m left ventriculography has recently been reported to be a useful method to assess LV diastolic dysfunction due to more stable parameters and because it is independent of the influence of systolic function, age and sex [7]. However, data on LV diastolic function in patients with HF are limited.

Left ventricle remodeling plays a critical role in the progression of HF. Several serum markers of cardiac extracellular matrix (ECM) have been shown to play an important role in myocardial remodeling in various types of heart disease in patients with HFrEF and in animal models [8] [9] [10]. Restrictive LV filling develops as a consequence of myocardial remodeling and cardiac fibrosis, and it has been reported to be closely associated with clinical outcomes [11, 12]. Detecting restrictive LV filling is therefore important, however it is highly dependent on the imaging tool.

Several cardiac ECM markers including type III aminoterminal propeptide of procollagen (PIIINP), matrix metalloproteinases (MMPs), and tissue inhibitor of metalloproteinase-1 (TIMP-1) have recently been shown to be of prognostic value in patients with HF [13] [14–17]. These markers reflect the collagen turnover process in the myocardium and are useful to evaluate fibrosis in the myocardium. Therefore, cardiac ECM markers could potentially be used to detect LV restrictive filling.

In this study, we investigated the relationships between serum markers of cardiac ECM and LV restrictive filling using Tc99m left ventriculography in patients with HFrEF.

## MATERIALS AND METHODS

### Patients

We studied 51 patients with chronic HF secondary to LV systolic dysfunction (LVEF ≤ 45% measured using Tc99m left ventriculography) who regularly visited the HF clinics at National Taiwan University Hospital. The clinical history of all patients was recorded, and they all received a full examination by a cardiologist. Demographic data including sex, age, current medications, functional status, and cardiovascular risk factors were recorded. Venous blood samples were collected in serum separation tubes after overnight fasting. After clotting and centrifugation, the serum was collected and stored at −60°C until analysis. In echocardiography, LVEF was measured via an apical 4-chamber view (area-length method) according to the procedures of the American Society of Echocardiography [18]. The study was approved by the Ethics Committee of National Taiwan University Hospital, and all of the patients provided written informed consent.

### Diastolic function assessment

We assessed LV diastolic function using Tc99m left ventriculography. Peak-filling rate (PFR) was defined as the greatest filling rate in early diastole, and corresponded to the peak value of the first derivative of the diastolic portion of the time-activity curve. The filling was normalized to end-diastolic volume (EDV), and thus the unit for PFR was EDV/s. The time to peak filling rate (TPFR), expressed in milliseconds, was defined as the interval between end-systole and PFR.

The normal PFR of the left ventricle has been reported to be 2.2 +/- 0.6 EDV/sec, and 198 +/- 22 ms for TPFR [14–17, 19]. By applying a 2-standard deviation cutoff point to the mean values of TPFR, the threshold for a restrictive pattern of LV filling was defined as a TPFR ≤ 154 ms.

### Laboratory analysis

Serum levels of brain natriuretic peptide (BNP) were measured using an enzyme immunoassay kit (BNP-32, Phoenix pharmaceuticals, Belmont, USA). The intra- and inter-assay variations were < 5% and < 14%, respectively. Serum levels of type I aminoterminal propeptide of procollagen (PINP) were measured using a rapid equilibrium radioimmunoassay kit (No. 67034, Orion Diagnostica, Espoo, Finland). The intra- and inter-assay variations were both < 7%, and the detection limit was 2 μg/l. Serum PIIINP was determined using a coated-tube radioimmunoassay method (No. 68570, Orion Diagnostica, Espoo, Finland). The intra- and inter-assay variations of serum PIIINP were both < 5%, and the detection limit was 0.3 μg/l. TIMP-1 was measured using an ELISA kit (DTM100, R & D Systems, Minneapolis, USA). The intra- and inter-assay variations of serum TIMP-1 were both < 5%, and the detection limit was 0.08 ng/ml. Serum MMP-2 was measured using an ELISA kit (DMP200, R & D Systems, Minneapolis, USA). The intra- and inter-assay variations of this method were < 6% and < 8%, respectively, with a detection limit of 0.16 ng/ml. MMP-9 was also measured using an ELISA kit (DMP900, R & D Systems, Minneapolis, USA). The detection limit was 0.156 ng/mL, and the intra- and inter-assay variations were < 3% and < 8%, respectively.

### Statistical analysis

Continuous data were expressed as median value and interquartile range. Comparisons between groups for continuous data were performed using the Mann-Whitney U-test, and differences between proportions were assessed using the chi-square or Fisher's exact test. Spearman's non-parametric correlation test was used to analyze associations between ventricular diastolic function and serum fibrosis markers. Linear regression analysis was used to examine associations between serum fibrosis markers and LV restrictive diastolic function. The serum cardiac ECM markers were log-transformed due to non-normality as detected using the Kolmogorov-Smirnov test before being entered into the linear regression model. To compare the ability of serum cardiac ECM markers to predict the patients with a restrictive pattern in diastolic filling (TPFR ≤ 154 ms), we used areas under the receiver operator characteristic (ROC) curve determined by logistic regression analysis. The optimal cutoff points of serum fibrosis markers were obtained from the Youden index.

We also used C-statistics to describe the discrimination of the models before and after adding serum cardiac ECM markers [20–22]. Net reclassification improvement (NRI) and integrated discrimination improvement (IDI) models were performed to assess improvements in risk prediction using two different logistic regression models [21]. All statistical analyses were performed using R software (http://www.r-project.org/) version 2.15.2. Statistical significance was set at p < 0.05.

## RESULTS

### Patient characteristics

Fifty-one (39 males and 12 females) patients were enrolled with a median age of 51.8 years, and median LVEF of 36.2% by echocardiography and 31.9% by Tc99m left ventriculography. Their median NYHA was 2, with 8, 27, 14, and 2 patients having a NYHA of I, II, III, and IV, respectively. The other demographic and biochemical data and medication history are shown in Table 1.

We divided the patients into two groups based on the presence of a restrictive pattern in Tc99m radionuclide ventriculography. The mean age was significantly older in the restrictive pattern group (p = 0.028), but there were no significant differences in the other demographic data (Table 1).

**Table 1:** Demographic data between patients of restrictive and non-restrictive diastolic function of LV

	Total population	Restrictive diastolic function (n=21)	Non-restrictive diastolic function (n=30)	*p*-value
Age	51.8 (47.2-73.0)	68.2 (48.4, 78.3)	50.4 (42.8, 64.8)	0.028
Gender, M/F	39 / 12	15/6	24/6	0.478
Body mass index, Kg/m2	25.1 (22.3, 29.0)	25.2 (21.9, 29.4)	25.0 (22.7, 29.0)	0.969
LVEF by echocardiography, %	36 (26, 41)	35(24, 42)	37 (26, 43)	0.730
NYHA functional classification, I/II/III/IV	8/27/14/2	3/10/7/1	5/17/7/1	0.861
Etiology for heart failure				
Ischemic	21 (41%)	9 (43%)	12 (40%)	0.838
Non-Ischemic	30 (59%)	12 (57%)	18 (60%)	
Co-morbidity				
Hypertension	22 (43%)	10 (48%)	12 (40%)	0.589
Diabetes Mellitus	13 (25%)	6 (29%)	7 (23%)	0.673
Atrial fibrillation	14 ( 27%)	7 (33%)	7 (23%)	0.431
Biochemistry				
Hemoglobin, g/dL	14.2 (12.9, 15.5)	13.9 (12.4, 15.6)	14.2 (13.3, 15.6)	0.406
Triglyceride, mg/dl	146 (85, 216)	112 (83, 179)	165 (87, 280)	0.203
Total cholesterol, mg/dl	156 (186, 212)	171 (156, 215)	199 (158, 212)	0.328
Fasting glucose, mg/dl	100 (88, 136)	100 (87, 135)	100 (90, 142)	0.762
Creatinine, mg/dL	1.2 (1.0, 1.4)	1.3 (1.1, 1.5)	1.2 (1.0, 1.4)	0.268
BNP, pg/mL	2061 (1430, 2520)	2263 (1413, 2780)	1957 (1575, 2435)	0.579
Medications				
ACEI / ARB	28 (55%)	10 (48%)	18 (60%)	0.382
β-blocker	34 (67%)	14 (67%)	20 (67%)	1.000
Loop agents	35 (69%)	17 (81%)	18 (60%)	0.112
Digoxin	28 (55%)	13 (62%)	15 (50%)	0.400
Spironolactone	20 (39%)	9 (43%)	11 (37%)	0.656
Statin	7 (14%)	3 (14%)	4 (13%)	0.923

Abbreviations: BNP= Brain natriuretic peptide; ACE-I= angiotensin converting enzyme inhibitor; ARB= angiotensin receptor blocker; LVEF= left ventricular ejection fraction; MMP = matrix metalloproteinase; NYHA Fc= New York Heart Association functional classification.

### Correlation between serum fibrosis markers and Tc99m radionuclide ventriculography parameters

The mean level of serum fibrosis markers and Tc99m radionuclide ventriculography are shown in Table 2. TPFR of the left ventricle was significantly correlated with serum levels of TIMP-1 (r = -0.337, p = 0.016), PIIINP (r = -0.445, p = 0.001), and MMP-2 (r = -0.391, p = 0.05), whereas TPFR of the right ventricle was significantly correlated with serum PIIINP levels (r = -0.320, p = 0.022) (Table 3).

**Table 2:** Median level of serum fibrosis markers and Tc99m radionuclide ventriculography parameter (n=51)

	Total	Restrictive diastolic function (n=21)	Non-restrictive diastolic function (n=30)	p-value
Serum fibrosis markers				
PINP, μg/l	33.02 (24.16-46.85)	40.48 (27.35, 51.83)	30.68 (22.05, 40.67)	0.112
TIMP-1 ng/ml	131.3 (96.6-181.8)	142.70 (119.95, 204.60)	114.7 (93.35, 155,35)	0.019
PIIINP, μg/l	6.4 (4.64-7.21)	7.03 (6.43, 9.60)	5.13 (4.29, 6.87)	0.002
MMP-2 ng/ml	248.47 (218.39-318.76)	310.49 (234.18, 419.05)	236.09 (209.03, 268.03)	0.002
MMP-9 ng/ml	58.1 (38.5-100.7)	52.70 (32.10, 104.50)	59.15 (39.77, 100.65)	0.688
Technetium-99m radionuclide ventriculography parameter				
LVEF, %	31.9 (21.7, 38.9)	24.9 (21.6, 31.5)	35.6 (21.9, 40.1)	0.121
LV-PFR, EDV/sec	1.50 (1.07, 1.88)	1.57 (1.22, 2.14)	1.44 (1.04, 1.79)	0.461
LV-TPFR, ms	177 (118, 348)	101 (64, 141)	250 (196, 416)	<0.001
RVEF, %	43.0 (31.0, 48.8)	42.3 (31.5, 47.3)	43.0 (30.5, 52.1)	0.660
RV-PFR, EDV/sec	2.06 (1.58, 2.80)	2.10 (1.71, 3.12)	1.95 (1.53, 2.54)	0.394
RV-TPFR, ms	145 (80, 308)	71 (59, 147)	222.5 (142, 399)	<0.001

Abbreviations: LVEF= left ventricular ejection fraction; LV-PFR= left ventricle-peak filling rate; LV-TPFR= left ventricle-time to peak filling rate; RVEF= right ventricular ejection fraction; RV-PFR= right ventricle-peak filling rate; RV-TPFR= right ventricle-time to peak filling rate; MMP = matrix metalloproteinase; NYHA Fc= New York Heart Association functional classification; PINP = type I aminoterminal propeptide of procollagen; PIIINP = type III aminoterminal propeptide of procollagen; TIMP = tissue inhibitor of metalloproteinase.

**Table 3:** Spearman rank correlation between the parameters of Tc99m radionuclide ventriculography & serum fibrosis markers

	RNA-LVEF	LV-PFR	LV-TPFR	RNA-RVEF	RV-PFR	RV-TPFR
PINP	r = -0.034 p = 0.813	r = 0.056 p = 0.696	r = -0.220 p = 0.121	r = -0.014 p = 0.920	r = 0.106 p = 0.461	r = -0.186 p = 0.191
PIIINP	r = 0.124 p = 0.387	r = 0.283 p = 0.044	r = -0.445 p = 0.001	r = -0.110 p = 0.441	r = 0.214 p = 0.131	r = -0.320 p = 0.022
TIMP-1	r = 0.109 p = 0.445	r = 0.138 p = 0.335	r = -0.337 p = 0.016	r = -0.066 p = 0.644	r = -0.029 p = 0.842	r = -0.179 p = 0.209
MMP-2	r = -0.049 p = 0.732	r = 0.099 p = 0.491	r = -0.391 p = 0.005	r = -0.210 p = 0.140	r = 0.077 p = 0.589	r = -0.231 p = 0.102
MMP-9	r = -0.001 p = 0.995	r = 0.216 p = 0.128	r = -0.041 p = 0.773	r = 0.013 p = 0.929	r = 0.154 p = 0.282	r = 0.066 p = 0.645

LVEF= left ventricular ejection fraction; LV-PFR= left ventricle-peak filling rate; Abbreviations: LV-TPFR= left ventricle-time to peak filling rate; RVEF= right ventricular ejection fraction; RV-PFR= right ventricle-peak filling rate; RV-TPFR= right ventricle-time to peak filling rate; MMP = matrix metalloproteinase; NYHA Fc= New York Heart Association functional classification; PINP = type I aminoterminal propeptide of procollagen; PIIINP = type III aminoterminal propeptide of procollagen; TIMP = tissue inhibitor of metalloproteinase.

### Relationship between the presence of a restrictive pattern in Tc99m radionuclide ventriculography and serum fibrosis markers

The patients with a restrictive LV pattern in Tc99m radionuclide ventriculography had significantly higher levels of serum fibrosis markers including TIMP-1 (p = 0.019), PIIINP (p = 0.002), and MMP-2 (p = 0.002) (Table 2). The relationship between serum fibrosis markers and the presence of a restrictive pattern were still significant after adjusting for age and sex (Table 4). The relationship between PIIINP or MMP-2 and the presence of a restrictive pattern remained significant after adjusting for age, sex, and serum creatinine level (Table 4).

**Table 4:** Logistic regression between serum fibrosis markers and left ventricle restrictive diastolic function

		log PINP	log PIIINP	log TIMP-1	log MMP-2	log MMP-9
Model 1	OR value 95% CI p value	14.440 0.658 – 316.812 0.090	436.115 5.328 - 35704 0.007	126.813 2.566 – 6268.181 0.015	3086.568 12.220-779604 0.004	1.162 0.206 – 6.563 0.865
Model 2	OR value 95% CI p value	18.928 0.606 – 591.478 0.094	622.391 5.168 – 74948 0.008	72.266 1.091 – 4784.690 0.045	1849.149 4.333 - 789163 0.015	3.025 0.412 – 22.233 0.277
Model 3	OR value 95% CI p value	13.776 0.284 - 668.015 0.185	6424.241 11.759 – 3509696 0.006	103.644 0.796 – 13503.066 0.062	2607.875 3.383 - 2151712 0.020	2.615 0.357 – 19.130 0.277

Model 1 unadjusted.

Model 2 adjusted for age and sex.

Model 3 adjusted for age, sex, and serum creatinine level.

Abbreviations: OR = odds ratio; CI = confidence interval; PINP = type I aminoterminal propeptide of procollagen; PIIINP = type III aminoterminal propeptide of procollagen; TIMP = tissue inhibitor of metalloproteinase.

### Prediction of a restrictive pattern in diastolic filling by various cardiac ECM markers and clinical parameters

**Figure 1:**
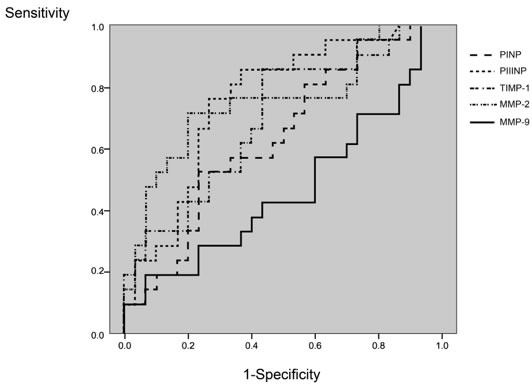
Analysis of the discrimination power of the two group by receiver operating characteristic curve analysis The areas under the curve of PINP, PIIINP, TIMP-1, MMP-2, and MMP-9 were 0.632, 0.758, 0.695, 0.751, 0.467, respectively.

To predict the patients with a restrictive pattern in diastolic filling (TPFR ≤ 154 ms), ROC analysis showed an area under the curve (AUC) of 0.632 for PINP, 0.758 for PIIINP, 0.695 for TIMP, 0.751 for MMP-2, and 0.467 for MMP-9 (Figure 1). Using a level of PIIINP of 6.22 mg/L as a cut-off point, the sensitivity, specificity, positive predictive rate, and negative predictive rate were 100%, 67%, 55%, and 100%, respectively. The AUC of clinical parameters and BNP were 0.631 for age, 0.471 for male gender, 0.595 for serum creatinine level, and 0.565 for BNP, respectively.

### Analysis of the predictive value of cardiac ECM markers by AUC, NRI and IDI models

We further examine the ability of the three cardiac ECM markers (PIIINP, TIMP-1, and MMP-2) to predict the patients with a restrictive pattern in diastolic filling after adding two clinical parameters (age and sex) or BNP. In C-statistics, all three cardiac ECM markers significantly increased the AUC of creatinine (Table 5). Furthermore, after adding cardiac ECM markers, the AUC of the new ROC curve was above 0.9 in all three models. The combination of creatinine and PIIINP had the highest AUC of 0.944. In the NRI and IDI models, PIIINP and MMP-2 significantly improved the predictive power of both clinical parameters and BNP, and TIMP-1 significantly improved the predictive power of creatinine (Table 5).

**Table 5:** Analysis of the predictive values of biomarkers by AUC, NRI, and IDI models

	AUC	AUC **p** value	NRI	NRI **p** value	IDI	IDI **p** value
PIIINP	0.758					
TIMP-1	0.695					
MMP-2	0.751					
Age	0.631					
+PIIINP	0.792	0.277	0.638	0.018	0.125	0.012
+TIMP-1	0.746	0.547	0.257	0.347	0.085	0.055
+MMP-2	0.774	0.371	0.810	0.002	0.112	0.017
Creatinine	0.595					
+PIIINP	0.944	0.003	1.124	<0.001	0.268	<0.001
+TIMP-1	0.901	0.015	0.771	0.002	0.151	0.008
+MMP-2	0.904	0.013	0.571	0.031	0.156	0.005
BNP	0.565					
+PIIINP	0.770	0.398	0.800	0.002	0.144	0.005
+TIMP-1	0.699	0.895	0.448	0.103	0.117	0.020
+MMP-2	0.737	0.617	0.676	0.011	0.180	0.003

BNP, Brain natriuretic peptide; LVEF, left ventricular ejection fraction; MMP, matrix metalloproteinase; PIIINP, type III aminoterminal propeptide of procollagen; TIMP, tissue inhibitor of metalloproteinase; AUC, area under the curve; NRI, Net reclassification improvement; IDI, integrated discrimination improvement.

## DISCUSSION

The major findings of this study are: 1) serum ECM markers including TIMP-1, PIIINP, and MMP-2 were significantly correlated to LV TPFR; 2) the patients with a restrictive pattern of LV diastolic function had higher TIMP-1, PIIINP, and MMP-2 levels; 3) the results were still significant after adjusting for age and sex; 4) cardiac ECM markers significantly improved the power of the clinical parameters to predict the patients with a restrictive pattern in diastolic filling; and 5) the combination of PIIINP and creatinine had greatest power (AUC 0.944) to predict the patients with a restrictive pattern in diastolic filling. These findings not only reconfirmed the association between LV restrictive filling and cardiac ECM turnover, but also showed the potential of cardiac ECM markers as a useful tool to detect LV restrictive filling.

ECM turnover regulates a fine balance between synthesis and degradation [10, 23], and altering the balance of ECM markers can lead to cardiac fibrosis [17]. Cardiac fibrosis provides substrate for arrhythmia which can lead to sudden cardiac death. In addition, the worsening of cardiac diastolic dysfunction can also result in the progression of HF [24]. Therefore, measuring serum ECM markers may be a simple and reliable method to evaluate the degree of cardiac fibrosis [24]. Furthermore, clinical studies have shown that cardiac ECM markers can provide useful information on clinical symptoms, outcomes, and arrhythmia in patients with HFrEF [25].

In the present study, PIIINP had the best AUC. Both collagen types I and III are present in normal and diseased myocardial tissue. PINP and PIIINP are by-products of collagen type I or III synthesis, and therefore they are considered to be markers of collagen biosynthesis [26]. PIIINP is more specific to tissue in patients with cardiac disease, even though type I collagen is more abundant in the myocardium [25]. This is probably the reason why PIIINP had a better AUC that PINP in our analysis. PIIINP has also shown to have a better association with clinical outcomes than PINP in other studies [27].

MMPs are a family of enzymes that contribute to extracellular remodeling in several disease states. In addition, a family of inhibitors called tissue inhibitors of MMPs (TIMPs) have been shown to exist and to tightly regulate MMP activity. TIMP-1 has been shown to be highly associated with collagen accumulation and cardiac diastolic function [28, 29]. Both MMP-2 and TIMP-1 have been demonstrated to have a prognostic role in patients with HF [30] [31]. In the present study, the AUC of MMP-2 was 0.751, which is very close to the AUC of PIIINP (0.758). We previously showed that MMP-2 was the best ECM marker to predict clinical outcomes [31]. Therefore, it is not surprising that MMP-2 was highly associated with LV restrictive filling in the present study.

In the Val-HeFT trial, a decrease in LVEF was associated with an increase in all-cause mortality at 23 months [32]. In addition, Solomon et al. reported a 39% increase in all-cause mortality in patients with HFrEF for every 10% reduction in ejection fraction below 45% [33]. However, relying on a univariate predictor such as LVEF to predict the prognosis is not sufficient, and patients with HFrEF and diastolic dysfunction have a lower survival rate. Both a deceleration time ≤ 140 ms and early to late flow velocity of mitral inflow > 1 mean have been shown to be powerful independent predictors of mortality according to studies which evaluated mitral inflow patterns by Doppler echocardiography [34] [35]. In addition, Temporelli et al. reported that after optimal medical treatment, the patients’ response to medication with reversal of the restrictive pattern at 6 months resulted in a lower cardiac mortality rate and fewer events requiring hospitalization than those with persistence of the restrictive pattern [36]. Patients with a persistent LV restrictive filling pattern have also been reported to have a significantly lower survival rate compare to patients with a reversible LV filling pattern [37].

Diastolic function can be estimated using echocardiography, cardiac MRI, and gate myocardial perfusion SPECT. Echocardiography is a simple, non-invasive and cost-effective tool, however alterations in loading conditions, operator technique, and the limitation of an acoustic window can all affect interpretation of the results. Although more expensive than echocardiography, cardiac MRI provides excellent spatial resolution and more precise parameters to assess diastolic function [38], however it takes time and needs the patient's cooperation. Moreover, patients with intra-cardiac devices are not candidates for MRI, and gadolinium injections to detect myocardial fibrosis may not be tolerable in patients with chronic kidney disease [38] [39].

Gated myocardial perfusion SPECT is widely used to assess perfusion abnormalities, LV systolic function and volume. First-pass and gated blood-pool scintigraphy including Tc99m left ventriculography are useful methods to assess LV diastolic dysfunction due to more stable parameters and independence from the influence of systolic function, age and sex [7]. Prolonged LV PFR and TPFR indicates impairment of diastolic function. After analyzing 90 patients with normal exercise gated myocardial perfusion SPECT, abnormal PFR and the threshold for abnormal TPFR were derived by 2 standard deviations to the mean values (PFR < 1.71 EDV/s and TPFR > 216.7 ms) [7]. Compared to PFR, TPFR appears to be a more useful parameter to assess diastolic function independently of systolic function, heart rate and age [7]. For an extremely short TPFR, however, no previous studies have assessed restrictive diastolic function by Tc99m left ventriculography. By applying a 2-standard deviation cutoff to the mean value of TPFR, we defined a TPFR < 154 ms as a restrictive pattern of LV diastolic function. In this study, TPFR was negatively correlated with cardiac ECM markers including PIIINP, MMP-2 and TIMP-1. In the patients with a LV restrictive pattern, the cardiac ECM markers were significantly elevated. Furthermore, using the correlations between cardiac serum markers and restrictive function, we determined the threshold of cardiac ECM markers. This suggests that it will be possible to detect restrictive LV filling in failing hearts in the near future through simple blood testing.

There are several limitations to this study. First, the number of enrolled patients was relatively small, and may not be sufficient to provide definitive evidence. However, our findings may be sufficient to prompt further research. Second, we focused on HFrEF, and we are not sure whether similar results would be found in patients with HF with preserved ejection fraction. This could form the basis of further studies. Third, no histological evidence was available to prove myocardial remodeling and to assess the extent of fibrosis. Elevated levels of serum cardiac ECM markers may not present to the same degree over the myocardium. However, without clinical indications, it is not ethical to perform endomyocardial biopsies in these patients. Fourth, there is currently no general consensus on the definition of LV diastolic dysfunction and restrictive filling pattern in radionuclide ventriculography. Therefore, we used our own definition of “restrictive pattern” in this study. Further large-scale clinical studies and consensuses are needed to define the criteria of diastolic dysfunction and restrictive pattern in radionuclide ventriculography. Finally, a longer period of follow-up may be necessary to ascertain the clinical prognostic value of each biomarker and to differentiate outcomes between the patients with and without a restrictive LV filling pattern.

In conclusion, PIINP, MMP-2, and TIMP-1 are significantly associated with a shortened TPFR in Tc99m left ventriculography and the presence of restrictive LV filling in patients with HFrEF. These serum markers have the potential to detect the presence of LV restriction in patients with HFrEF.
